# Tricuspid Valve Repair with Autologous Pericardium in a Patient with Infective Endocarditis

**DOI:** 10.21470/1678-9741-2019-0287

**Published:** 2021

**Authors:** Henry Leonardo Robayo Amórtegui, Javier Páez Cristancho, Igor Donís-Gómez

**Affiliations:** 1 Colsubsidio Investiga Research Group, Clínica Infantil de Colsubsidio, Bogotá, Colombia.; 2 Pediatric Cardiovascular Surgery Department, Clínica Infantil de Colsubsidio, Bogotá Colombia.

**Keywords:** Tricuspid Valve, Child, *Corynebacterium Diphtheriae*, Endocarditis, Bacteria, Cardiac Surgical Procedures, Pericardium

## Abstract

Infective endocarditis is a rather uncommon disease, but it has significant mortality rates in the pediatric population (5% to 10%). We report a case of an infant patient with multiple vegetation in the tricuspid valve secondary to infective endocarditis caused by *Corynebacterium diphtheriae*. A tricuspid valvuloplasty was performed with a fenestrated autologous pericardium patch, providing satisfactory outcomes. This technique is simple, innovative, effective, and it could be applied in similar cases.

**Table:** 

Abbreviations, acronyms & symbols	
IE	= Infective endocarditis
NV	= Neovalve
RA	= Right atrium

## INTRODUCTION

Five to 36% of infective endocarditis (IE) cases involve the tricuspid valve^[1]^. With an incidence of 0.05 cases to 0.12 cases per 1,000 habitants, IE is a rather uncommon disease in the pediatric population. Its high risk of mortality rates between 5% and 10%. Thus, it is necessary to lead multidisciplinary management in order to achieve an accurate diagnosis and early surgical treatment^[2-4]^. The objective of this report is to inform about a tricuspid valve replacement using a fenestrated autologous pericardium patch performed in a pediatric patient with multiple vegetation in the tricuspid valve.

## CASE REPORT

A three-year-old male patient was admitted with a two-month history of abdominal pain, intermittent fever, asthenia, and adynamia. During physical examination, he presented jaundice, II/IV tricuspid murmur, and generalized swelling. Blood cultures isolated *Corynebacterium diphtheriae*. Echocardiography revealed a deformed and enlarged tricuspid valve with multiple vegetation in both anterior and posterior leaflets. One of them was as big as to prolapse the right ventricle, as it had a 12 mm diameter and III/IV grade insufficiency with pulmonary systolic pressure of 42 mmHg.

The pediatric cardiovascular surgical service opted for performing urgent surgical intervention, given the sepsis persistence and risk for an embolic event due to the vegetation. Surgery was performed in extracorporeal circulation with low hypothermia (32ºC), conducting bypass with Alpha-Stat. Arterial cannulation of ascending aorta and both cavae was done. Del Nido blood-based cardioplegia was utilized for heart arrest. The valve was found to be completely compromised by the infectious process, thus it had to be resected and samples were sent for further examination.

**Fig. 1:**
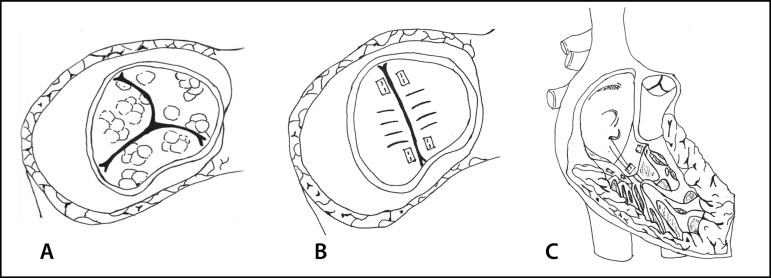
(A) Vegetations on the tricuspid valve. (B) Neovalve with two rectangular segments of autologous pericardium with four fenestrations. (C) Neovalve patches were tacked into the papillary muscles with separated sutures, then in the tricuspid annulus with continuous suture.

**Fig. 2:**
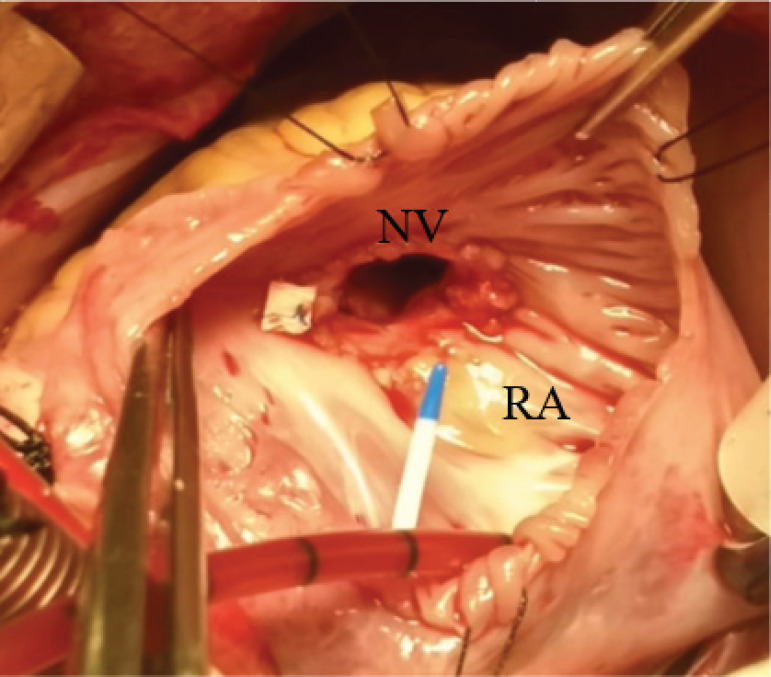
Photograph of surgical repair of the tricuspid valve with an autologous pericardium neovalve (NV). RA=right atrium

A tricuspid neovalve was tailored with two rectangular segments of autologous pericardium. It measured approximately half of the tricuspid annulus in width and 1.3 times the distance between papillary muscles and valvular annulus in length. The patches were tacked into the papillary muscles with separated polypropylene 5/0 sutures, then in the tricuspid annulus with a 6/0 polypropylene continuous suture (Figures 1 and 2). To avoid stenosis and promote ventricular filling, four fenestrations as big as 1/3 of the length of the patches were made in the inferior part of both patches. A cold saline solution delivered through the right ventricle demonstrated a competent tricuspid valve.

**Fig. 3:**
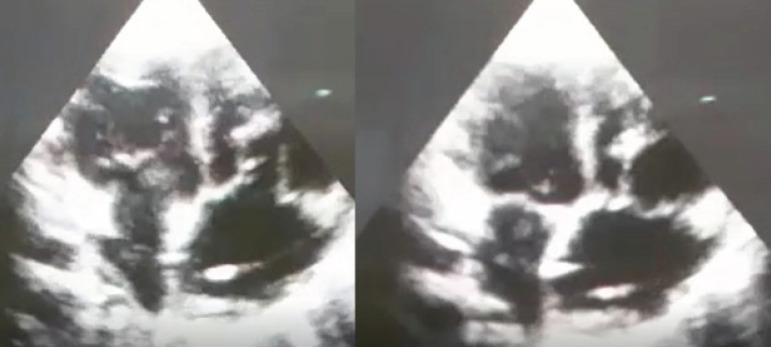
Postsurgical echocardiogram of the replacement of the tricuspid valve with an autologous pericardium.

Once the repair was finished, clamp was removed from the aorta. No further rhythm dysfunction ensued, and hemodynamic state was positive. After two weeks of surgery, neither neurological nor cardiovascular complications occurred. An echocardiogram revealed a well-functioning neoprosthesis of autologous pericardium, as well as a satisfactory biventricular function with no presence of abscess or vegetations (Figure 3). No sepsis or cardiac failure were detected on follow-up after a 12-week postoperative course.

## DISCUSSION

IE involving the tricuspid valve caused by *Corynebacterium diphtheriae* is rarely identified among pediatric population^[3]^. Endothelial damage is caused by two mechanisms, one being a direct damage to the endothelial surface from an external agent, the other being an indirect damage, in which an external agent interferes with normal functioning of the tricuspid valve^[2,4]^. Currently, there are no guides or recommendations concerning the surgical management of pediatric patients with IE^[2]^.

Nonetheless, the Society of Thoracic Surgeons recommends in their 2011 guide surgical intervention for the tricuspid valve in patients with IE who present persistent sepsis and severe tricuspid regurgitation^[5]^.

Shamszad et al.^[6]^ inform that children might successfully undergo early tricuspid valve surgery with low complication rates, no need for reintervention, and lower mortality rates compared with the group which was indicated for medical management. In terms of the surgical technique, valvular replacement with biological or mechanical prostheses is recommended^[3]^. However, utilizing these valves brings complications, such as risk of bleeding, reintervention, embolism, and endocarditis^[7]^. In our case, we decided to perform complete reconstruction of the tricuspid valve with an autologous pericardium, which had fenestrations to ease ventricular filling and avoid increase in transvalvular gradient.

Theoretically, using an autologous pericardium would allow the growth of the neovalve, since it is made from autograph tissue. Nevertheless, strict follow-up for a longer period than two weeks is vital to determine whether any structural changes seem to appear with time. There have been reports of the use of tricuspid neovalves made from biological extracellular matrix tissue (Cormatrix) with a cylindrical shape. Like our technique, they were sutured into the papillary muscles^[8]^. However, fenestration of the inferior part of the neovalves in order to ease ventricular filling has yet to be reported.

Early surgical repair of the tricuspid valve must be the first treatment option in pediatric population with IE with persistence of sepsis, severe valvular insufficiency, vegetation presence, and ventricular dysfunction. Using fenestrated autologous pericardium for repair of the tricuspid valve has proven to be a successful method, which might be feasible for patients with complete valvular damage, even in adult population with extense valvular damage.

**Table:** 

Author's roles & responsibilities	
HLRA	Substantial contributions to the conception or design of the work; or the acquisition, analysis, or interpretation of data for the work; drafting the work or revising it critically for important intellectual content; final approval of the version to be published
JPC	Substantial contributions to the conception or design of the work; or the acquisition, analysis, or interpretation of data for the work; drafting the work or revising it critically for important intellectual content; final approval of the version to be published
IDG	Substantial contributions to the conception or design of the work; or the acquisition, analysis, or interpretation of data for the work; drafting the work or revising it critically for important intellectual content; final approval of the version to be published
